# Obesity-induced ocular changes in children and adolescents: A review

**DOI:** 10.3389/fped.2023.1133965

**Published:** 2023-03-23

**Authors:** Julia Dezor-Garus, Elżbieta Niechciał, Andrzej Kędzia, Anna Gotz-Więckowska

**Affiliations:** ^1^ Department of Ophthalmology, Poznan University of Medical Sciences, Poznan, Poland; ^2^ Department of Pediatric Diabetes, Clinical Auxology and Obesity, Poznan University of Medical Sciences, Poznan, Poland

**Keywords:** childhood obesity, ocular signs, retinal microvasculature, intraocular pressure, retinal nerve fiber layer, optical coherence tomography

## Abstract

Childhood obesity has reached epidemic levels worldwide. Overweight and obesity is associated with an increase in several inflammatory markers, leading to chronic low-grade inflammation responsible for macro- and microvascular dysfunction. While the impact of obesity on overall health is well-described, less is known about its ocular manifestations. Still, there are few studies in children and adolescents in this regard and they are inconsistent. However, some evidence suggests a significant role of overnutrition in the development of changes in retinal microvasculature parameters (wider venules, narrower arterioles, lower arteriovenous ratio). Higher values of intraocular pressure were found to be positively correlated with high body mass index (BMI) as well as obesity. In addition, the retinal nerve fiber layer (RNFL) values seem to be lower in obese children, and there is a significant negative correlation between RNFL values and anthropometric and/or metabolic parameters. Changes also could be present in macular retinal thickness and choroidal thickness as well as in the retinal vessel density in children with obesity. However, these associations were not consistently documented. The purpose of this review is to present the most current issues on child obesity and the related potential ocular effects through an overview of international publications from the years 1992–2022.

## Introduction

Childhood obesity has become one of the most important public health concerns. Over the past 50 years, its prevalence has increased globally, reaching pandemic levels. The World Health Organization (WHO) estimates that worldwide, over 340 million children and adolescents aged 5–19 years and almost 40 million children under the age of 5 years were overweight or obese in 2016 and 2020, respectively (1, 2). The latest data from the United States Centers for Disease Control and Prevention (CDC) has shown that the prevalence of obesity among United States children and adolescents was 19.3% in 2017–2018 (3). In addition, the COVID-19 pandemic worsened the burden of childhood obesity (4, 5). For example, in the United States, during the first year of the pandemic, the prevalence of obesity in children aged 5–11 years rose dramatically from 19% to 26% (5).

Obesity has emerged as a complex phenotype resulting from the interaction of genes, environment, and lifestyle (6). There are numerous factors contributing to childhood obesity, including socioeconomic aspects, psychosocial stress, gender, biology, physical inactivity, or a caloric intake that is greater than needs (7). Endocrine causes of pediatric obesity are identified in less than 1% of all cases (8).

Regardless of its cause, obesity results in the abnormal or excessive accumulation of fat. Adipose tissue, in addition to providing a reservoir of energy, serves as a major endocrine organ that secretes adipokines, growth factors, cytokines, and chemokines (9, 10). Increased proinflammatory cytokine secretion from adipose tissue and the infiltration of leukocytes, including macrophages, into the adipose tissue is responsible for chronic low-grade inflammation in obese individuals. This state impairs important functions in terms of regulating fatty acid oxidation, glucose and fat homeostasis, and insulin signaling. Oxidative stress, inflammation, and dyslipidemia accompanied by insulin resistance lead to micro- and macroangiopathy (11, 12).

**Figure 1:**
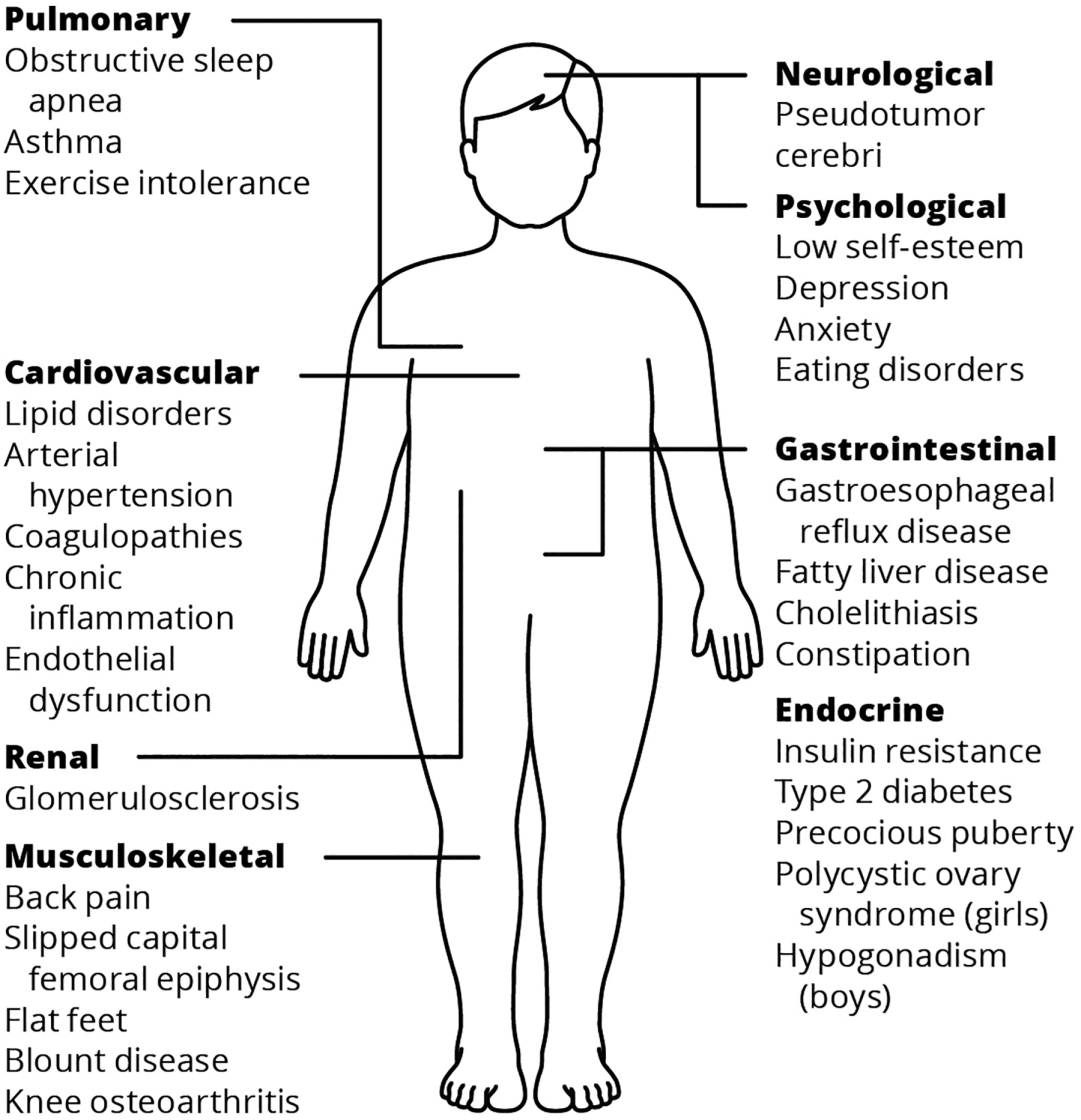
Systematic complications of childhood obesity (**7**, **13**, **14**).

Obesity can adversely affect health, and obesity-related diseases in children and adolescents are well-characterized (7, 13, 14) (shown in Figure 1).

Less is known about the potential ocular effects of childhood obesity. In adults, obesity has been linked to age-related cataracts, glaucoma, age-related maculopathy, and diabetic retinopathy (15). This review examines the potential ocular effects of childhood obesity.

### Data sources and searches

A literature search on PubMed and Google Scholar was performed using the following terms in various combinations: childhood obesity, obesity, children and adolescents, pediatric population, insulin resistance, retinal microvasculature, optical coherence tomography, optical coherence tomography angiography, foveal avascular zone, choroidal thickness, retinal nerve fiber layer, and intraocular pressure. All articles published between November 1992 and November 2022 were checked by title, abstract, and full text. Relevant articles are reviewed and summarized here.

### Changes in retinal vessel morphology in terms of childhood obesity

Since the 1990s, fundus photography has been widely used to evaluate the retinal vascular caliber. Hubbard et al. developed formulas to calculate the central retinal arteriolar equivalent (CRAE) and the central retinal venular equivalent (CRVE) calibers, as well as the dimensionless quotient arteriovenous ratio (AVR) (16, 17). Changes in retinal vessel caliber, particularly wider retinal venules and narrower retinal arterioles, have been found to increase the risk of cardiovascular disease in obese youth (18, 19). Obesity may have a profound effect on retinal vascular caliber. Increased adiposity and sedentary lifestyle behaviors negatively correlate with vessel width parameters. Growing evidence suggests that wider venules, narrower arterioles, and a lower AVR are related to a greater body mass index (BMI), larger waist circumference, and higher levels of sedentary behavior. In contrast, moderate-to-vigorous-intensity physical activity was associated with larger arterioles, narrower venules, and a higher AVR (20). Furthermore, other data demonstrated a strong relationship between retinal vascular calibers and obesity-induced hypertension. Retinal arteriolar narrowing, but not venular dilation, has been linked to increased systematic blood pressure (21). Interestingly, in a study conducted by Rijsk et al., in a multiple linear regression, a higher diastolic blood pressure (DBP) z-score and lower fasting glucose levels significantly contributed to a narrower retinal arteriolar diameter (22).

Undoubtedly, obesity has an impact on retinal vessel diameter; however, less data is available concerning the relationship between retinal vessel morphology and particular metabolic parameters. Leptin is secreted by adipocytes in proportion to body fat mass and plays a key role in energy homeostasis. Obesity can lead to leptin resistance and subsequent high leptin concentrations (23). Some studies have provided evidence in support of a positive association between leptin and retinal vessel diameter (24, 25). High levels of plasma leptin, dependent on BMI, have been associated with both wider CRVE (*p* = 0.032) and lower AVR (*p* = 0.010) (24). This is consistent with findings reported by Van Aart et al., indicating a positive significant relationship between zLeptin and CRAE (standardized coefficient *β *= 0.14) (25).

Apart from leptin, elevated levels of C-reactive protein (CRP), found in obese individuals, have also been linked to changes in retinal vessel diameter (26, 27). CRP is used as a clinical marker of inflammation, with elevated serum levels being a strong independent predictor of future adverse cardiovascular events (28). Hanssen et al. have reported that wider venular diameters were independently associated with higher levels of high-sensitivity C-reactive protein (hsCRP). The highest absolute diameter differences (+11.83um) were seen in retinal venules for each unit increase of hsCRP (*p* < 0.01) (26). Likewise, Gishti et al. showed that a higher CRP level is related to a wider venular caliber (difference 0.10 standard deviation score (SDS) (95% CI 0.06,0.14) per SDS increase in CRP), but this was not influenced by BMI (27).

Dyslipidemia, insulin resistance, and non-alcoholic fatty liver disease (NAFLD), other common features in obese children, also affect the retinal microvasculature. Some data have shown a positive relationship between hepatic fibrosis in pediatric NAFLD patients and the degree of retinopathy signs. In addition, obese and overweight children with retinopathy had significantly higher values of triglycerides (90.20 vs. 105.57 mg/dl, *p* = 0.04), basal insulin (12.97 vs. 17.20 mUI/ml *p* = 0.02), and HOMA-IR (2,76 vs. 3,37, *p* = 0.04) than patients without retinopathy (29).

Insulin-like growth factor 1 (IGF-I) plays a significant part in retinal vascularization. Recent data has demonstrated that overweight/obese children with retinopathy had significantly higher insulin-like growth factor 1 (IGF-1) SDS (-0.66 ± 0.9 vs. 0.03 ± 1.3, *p* = 0.04) compared to overweight/obese individuals without retinopathy (30). This suggests that high levels of IGF-1 in overweight/obese children and adolescents may be a potential contributing factor to microvascular damage.

However, the mechanisms linking childhood obesity to changes in retinal vessel morphology are not fully explained. It is hypothesized that increased adiposity is associated with the systemic microvasculature abnormalities that precede the development of overt disease. Microvascular processes may play a role in the pathogenesis of alterations in retinal vessel caliber, possibly through hyperinsulinemia or inflammatory pathways. Research has shown that arterial narrowing and venular widening are more evident among those with high levels of inflammatory and endothelial dysfunction markers (31). In addition, leptin has been demonstrated to directly impair endothelium-dependent vasodilation (32). Lastly, retinal venular dilatation may reflect a regulatory response to the increased total blood volume presented in obese children (31). However, further studies need to be carried out to clarify changes in retinal vessel caliber in obese children and adolescents.

### Influence of childhood obesity on intraocular pressure

Elevated intraocular pressure (IOP) may irreversibly damage the optic nerve and could result in visual impairment and blindness (33). Many studies have supported the association between higher values of intraocular pressure and high BMI (34, 35) as well as obesity (36–38). Akinci et al. have reported that the average IOP was significantly higher in obese children than in the control group; gender and age connections were not statistically significant between groups. In addition, diurnal fluctuations in IOP were also higher in the obese group (5.4 ± 1.8 mmHg) compared to the control group (2.8 ± 0.9 mmHg, *p* < 0.001). These findings show that BMI is an independent risk factor for elevated IOP in children and adolescents (38). The Shandong Children Eye Study produced similar results, indicating that higher values of IOP were positively correlated with increased body mass (*p* < 0.001) and BMI (*p* < 0.001) (34). Pileggi et al. demonstrated that elevated IOP was related to high BP in overweight/obese children. This suggests that obesity confers an increased risk for both elevated IOP and systemic vascular abnormalities, such as elevated BP and hypertension among children and adolescents (35). However, it is worth mentioning that several studies have not confirmed an association between obesity and changes in eye pressure among children and adolescents (39–43). The mechanisms underlying the association between IOP and obesity are yet to be fully established. However, it has been suggested that elevated IOP may be caused by excess intraorbital fat, increased episcleral venous pressure, and decreased aqueous outflow. Also, increased blood viscosity, present in obese individuals, can lead to an increase in outflow resistance in the episcleral veins. Finally, obesity-induced hypertension may influence IOP through increased pressure in the ciliary arteries, leading to excessive aqueous filtration (38, 44).

### Retinal nerve fiber layer thickness in the obese pediatric population

The retinal nerve fiber layer (RNFL) which is a sensitive structure, can be damaged due to a chronic inflammatory process. Optical coherence tomography (OCT) provides an objective and quantitative measurement of RNFL thickness.

Changes in retinal nerve fiber layer thickness are an important sign of early retinal damage. Therefore, there is increasing attention on RNFL thickness in obesity. Recent studies suggest that the RNFL is significantly thinner in obese children (37, 41, 42, 45, 46) Compared to healthy controls, obese individuals were characterized by binocular RNFL thickness asymmetry (41), decreased RNFL values in particular sectors (41, 42, 45), and thinner ganglion cell-inner plexiform layers (GCL + IPL) (41, 46). Moreover, changes in RNFL thickness are related to the degree of obesity. In children with severe obesity (SDS-BMI >4), RNFL thickness on average and in the inferior, superior and nasal quadrants were lower compared to overweight (SDS-BMI >1 to ≤2) and obese (SDS-BMI >2 to ≤4) individuals or healthy controls (SDS-BMI from −1 to 1) (42). The RNFL and GCL + IPL values for each sector are summarized in Tables 1, 2, respectively.

**Table 1:** Summary of reported values of OCT RNFL in obese children and adolescents (mean±) or median (min-max) in literature.

Reference	OCT device	*N*-number of patients/eyes	RNFL (um) Inferior quadrant	RNFL (um) Superior quadrant	RNFL (um) Nasal quadrant	RNFL (um) Temporal quadrant
**Pekel et al.** (**41**)	Zeiss Cirrus	O 41 C 41	128.2 ± 15.6 131.4 ± 18.4	121.6 ± 15.2[Table-fn table-fn2] 128.9 ± 16.2[Table-fn table-fn2]	72.4 ± 11.6 69.8 ± 11.4	66.9 ± 10.8 69.2 ± 9.3
**Karti et al.** (**46**)	Zeiss Cirrus	O 54/108 C 33/66	**122.4 ** **± 16.5** [Table-fn table-fn2] **131.9 ± 12.7** [Table-fn table-fn2]	**117.2 ± 15.2** [Table-fn table-fn2] **127.5 ± 15.3** [Table-fn table-fn2]	**72.3 ± 12.4** [Table-fn table-fn2] **80.4 ± 10.4** [Table-fn table-fn2]	**65.2 ± 11.8** [Table-fn table-fn2] **72.1 ± 10.0** [Table-fn table-fn2]
**Koca et al** (**45**)	Zeiss Cirrus	O 63/125 C 62/122	**122.9 ± 17.1** [Table-fn table-fn2] **127.2 ± 16.5** [Table-fn table-fn2]	**122.3 ± 14.8** **123.1 ± 16.8**	**68.7 ± 14.7** **69.9 ± 13.1**	**65.0 ± 8.5** [Table-fn table-fn2] **69.8 ± 10.3** [Table-fn table-fn2]
**Demir et al.** (**47**)	Zeiss Cirrus	O 85 C 30	127.53 ± 18.84 124.05 ± 17.75	123.64 ± 15.07 125.90 ± 13.39	70.51 ± 10.26 73.00 ± 12.95	68.13 ± 10.84 67.00 ± 8.57
**Özen et al.** (**48**)	Spectralis	O 38/76 C 40/80	121.2 ± 25.2 129.7 ± 23.0	120.1 ± 21.3 124.6 ± 19.7	71.3 ± 14.5 73.1 ± 14.1	74.6 ± 19.1 76.0 ± 10.7
**Kurtul et al.** (**49**)	Avanti Optovue	O 25/50 C 18/36	150.0 (142.0–159.5) 158.5 (117.0–173.0)	147.0 (130.5–154.0) 152.5 (137.0–166.0)	106.5 (96.0–118.5) 108.0 (93.0–118.0)	76.5 (72.0–82.0) 80.5 (73.0–87.0)
**Pacheco-Cervera et al.** (**42**)	Zeiss Cirrus	Ov 11 Ob. 42 S.O. 13 C 31	**139.2 ± 15.2** [Table-fn table-fn2] **130.7 ± 19.8** [Table-fn table-fn2] **117.8 ± 17.5** [Table-fn table-fn2] **131.3 ± 18.1** [Table-fn table-fn2]	**134.6 ± 14.5** [Table-fn table-fn2] **126.1 ± 18.7** [Table-fn table-fn2] **114.9 ± 21.3** [Table-fn table-fn2] **128.4 ± 20.6** [Table-fn table-fn2]	**72.6 ± 12.4** [Table-fn table-fn2] **72.3 ± 11.2** [Table-fn table-fn2] **60.7 ± 10.3** [Table-fn table-fn2] **75.2 ± 13.3** [Table-fn table-fn2]	70.8 ± 9.9 69.8 ± 16.3 67.5 ± 13.6 67.1 ± 12.8

OCT-optical coherence tomography; RNFL-retinal nerve fiber layer; O-obese and overweight group, C-control group, Ov-overweight group, Ob-obese group S.O.-severe obesity group.

* *p* < 0.05.

**Table 2:** Summary of reported values of OCT GCL + IPL in obese children and adolescents (mean ± SD) in literature.

Reference	OCT device	*N*-number of patients/eyes	GCL + IPL (um)inferior sector	GCL + IPL(um) Inferonasal sector	GCL + IPL(um) Inferotemporal sector	GCL + IPL(um) Superior sector	GCL + IPL(um) Superonasal sector	GCL + IPL(um) Superotemporal sector
**Pekel et al.** (**41**)	Zeiss Cirrus	O 41 C 41	82.2 ± 6.3 84.4 ± 6.7	84.2 ± 6.5 86.4 ± 6.8	82.8 ± 6.1 85.0 ± 5.9	84.1 ± 6.4 86.8 ± 6.4	85.5 ± 6.2 87.1 ± 6.2	**81.4 ± 5.8** [Table-fn table-fn4] **84.1 ± 5.9** [Table-fn table-fn4]
**Karti et al.** (**46**)	Zeiss Cirrus	O 54/108 C 33/66	**83.3 ± 6.9** [Table-fn table-fn4] **85.4 ± 5.8** [Table-fn table-fn4]	84.8 ± 7.6 86.8 ± 6.2	84.2 ± 6.7 85.6 ± 5.6	84.5 ± 7.0 85.8 ± 6.0	**85.1 ± 6.9** [Table-fn table-fn4] **87.9 ± 5.6** [Table-fn table-fn4]	83.9 ± 6.1 85.0 ± 5.7
**Demir et al.** (**47**)	Zeiss Cirrus	O 85 C 30	84.9 ± 5.8 84.7 ± 64	85.9 ± 5.9 85.6 ± 7.3	84.3 ± 5.7 84.9 ± 6.1	– –	85.6 ± 6.1 85.5 ± 8.3	84.9 ± 5.9 84.8 ± 6.6

OCT-optical coherence tomography; GCL-ganglion cell layer; IPL-inner plexiform layer; O-overweight and obese group, C-control group.

* *p* < 0.05.

In addition, metabolic parameters such as insulin, triglyceride, Homeostatic Model Assessment of Insulin Resistance (HOMA-IR), low-density lipoprotein (LDL), interleukin 6 (IL-6), and leptin have also been found to be negatively related to RNFL thickness (37, 41, 42, 45, 46, 48) (Table 3).

**Table 3:** Reported significant correlation between RNFL and anthropometric and metabolic parameters in literature.

Reference	Metabolic/anthropometric parameter	group	*r*	*p*
**Pekel et al.** (**41**)	mean BMI HOMA-IR Fasting insulin	O O O	−0,33 −0,34 −0,33	0.03 0.035 0.04
**Baran et al.** (**37**)	BMI-SDS WHR	O + C O + C	−0,203 −0,256	0.044 0.015
**Özen et al.** (**48**)	BMI-SDS DBP HOMA-IR LDL	O C O O O	−0,355 −0,345 −0,366 −0,394 −0,374	0.022 0.029 0.024 0.016 0.022
**Karti et al.** (**46**)	BMI-SDS Height-SDS Insulin HOMA-IR triglycerides	O + C O + C O + C O + C O + C	−0,386 −0,157 −0,229 −0,188 −0,301	<0.0001 0.038 0.002 0.013 <0.0001
**Pacheco-Cervera et al.** (**42**)	BMI-SDS Il-6 Leptin Fat mass index	O + C O O + C O O + C O O	−0.210 −0,302 −0,206 −0,274 −0,215 −0,276 −0,281	0.042 0.016 0.046 0.030 0.038 0.029 0.026

RNFL-retinal nerve fiber layer; BMI-body mass index; HOMA-IR Homeostatic Model Assessment for Insulin Resistance; SDS-standard deviation score-; WHR-waist- to-hip ratio; DBP-diastolic blood pressure; LDL-low-density lipoprotein; Il-6- interleukin 6; O- obese and overweight; C-control group.

Although many studies have shown that obesity is associated with changes in RNFL thickness, some studies have failed to show this relationship (47–49). The reason for changes in RNFL thickness in obese children is unclear. Increased resistance in the small vessels feeding the optic nerve head may result in temporary or permanent ischemia. Therefore, insufficient vascular supply of the optic nerve head as a result of endothelial dysfunction and autonomic changes creates conditions that damage nerve fibers. Also, it has been suggested that RNFL values decrease with an increase in inflammatory mediators (41).

### The association between choroidal thickness and macular retinal thickness in obese children

The choroid is one of the most highly vascularized tissues of the body, as it supplies the outer retina with nutrients and maintains the temperature and volume of the eye (50) Being the most vascular tissue in the eye, the choroid has been implicated in the pathophysiology of a variety of ocular diseases. Thinning of the choroid could be a predictive factor for retina damage.

The literature shows that changes in choroidal thickness (CT) may be induced by obesity. Several studies showed that children with obesity had thinner CT in comparison to the control group (36, 51, 52). Interestingly CT measurements were thinner among those obese patients who did not have IR as compared to those with IR (36, 51). The authors found a positive correlation between CT and HOMA-IR (36, 51). Differences in CT values in obese with and without IR can be explained by the vascular action of insulin, which stimulates endothelial production of NO with subsequent choroidal vasodilation and increased CT (53).

Obesity not only affects CT but also macular retinal thickness (MRT) and macular retinal volume (MRV). Some studies have reported that these ocular parameters are significantly decreased in obese children (52, 54). In one study, MRT and MRV values tended to be thinner with an increase in BMI-SDS and waist circumference standard deviation score (WC-SDS) (54).

However, a clear link between obesity and decreased CT and MRT has not yet been established. In a study conducted by Bulus et al., CT had higher values in obese patients, moreover, CT was positively correlated with BMI, BMI-SDS, Weight-SDS, obesity duration, and cholesterol level (40). Likewise, Kurtul et al. found a higher MRT in obese patients (49). A positive correlation between body fat percentage and CT has also been demonstrated (37).

A strong inflammatory response in adipose tissue, leading to oxidative stress and hypoxia, may cause changes in CT in obese individuals (40). In addition, thinning of the choroidal layer could result from the autonomic regulation of choroidal blood flow. Decreased levels of NO, as a direct result of obesity, lead to a reduction in choroidal blood flow and subsequent choroidal thickness (55).

Interestingly, it has been shown that weight loss may have a positive effect on ocular structures. A study investigating the effect of rapid BMI decrease with bariatric surgery in obese adults showed improvements in CT values after weight loss. The favorable effect of weight loss might be related to improvements in the autoregulation of choroidal blood flow and a decrease in medial arterial pressure (56).

However, it must be emphasized that some studies have not found a significant difference between choroidal and retinal thickness in obese children compared to healthy controls (37, 40, 57). Further studies to verify changes in CT and MRT are needed in obese juvenile populations.

### Influence of childhood obesity on retinal vascular density measured by optical coherence tomography angiography

Optical coherence tomography angiography (OCTA) is a novel technique that allows for a more accurate evaluation of the retinal vasculature, with the ability to isolate particular vascular plexuses (superficial and deep plexuses). OCTA has become an indispensable tool used in diagnosing and monitoring retinal diseases, for example choroidal neovascularization (CNV) in the course of age-related macular degeneration and high myopia or the estimation of foveal avascular zone (FAZ) size in the course of diabetic retinopathy, sickle cell-related retinopathy, or retinal vascular occlusion diseases.

So far, there have been only a few studies that used OCTA to evaluate retinal vessel density in the obese pediatric population. However, some of these studies have suggested that childhood obesity may affect the retinal microvasculature. Kurtul et al. and Han et al. showed that superficial, as well as deep capillary plexuses in the foveal area were larger in obese children in comparison to age- and sex-matched healthy controls (49, 58). Likewise, Dereli et al. reported that significantly greater vessel density in the parafoveal area of the superficial capillary plexus was observed in children with obesity (59). Noteworthy Han et al. reported lower vessel density in the inferior and nasal parafovea and temporal perifovea of deep capillary plexus in obese children. There is still no objective explanation for changes in the vascular density of retinal plexuses in childhood obesity. It has been suggested that pro-inflammatory cytokines may lead to degenerative changes (59). However, Kurtul et al. advocate that increased total body mass (with increased intra- and extracellular water), bone, and skeletal muscle mass may play a central role in elevated OCTA values (49).

Still, differences in OCTA values between obese and healthy children were not demonstrated by all authors (57), emphasizing the need for further research.

## Conclusion

Obesity is one of the most serious global public health challenges; however, its impact on ocular health in children is still not well-studied. To the best of our knowledge, this is the first review to examine the potential ocular effects of childhood obesity. Similar comparisons have only been made in the adult population. According to the findings of this review, the majority of studies found a significant association between adiposity and changes in vessel width parameters (wider venules, narrower arterioles, lower arteriovenous ratio). Many articles showed a positive relation between higher values of intraocular pressure and high BMI as well as obesity. In most studies, the retinal nerve fiber layer (RNFL) values were lower in obese children, and a significant negative correlation was found between RNFL values and anthropometric and/or metabolic parameters. Changes were also present in macular retinal thickness and choroidal thickness as well as in the retinal vessel density in children with obesity. However, these associations have not been consistently documented. In addition, there is a lack of studies underlying these eye changes in obese pediatric patients. The inconsistency of the results and an insufficient explanation for the observed changes suggest that further investigations are required to clarify these associations. The effectiveness of obesity management in reducing the risk of eye alterations is also unknown, but research in this area might provide significant insight into the potential use of weight loss strategies to reduce the burden of eye diseases in children with obesity. Moreover, studies examining the effects of a potentially anti-inflammatory nutritional intervention reducing obesity-induced ocular changes might deliver valuable and practical information.

## References

[1] Bentham J Di Cesare M Bilano V Bixby H Zhou B Stevens GA Worldwide trends in body-mass index, underweight, overweight, and obesity from 1975 to 2016: a pooled analysis of 2416 population-based measurement studies in 128·9 million children, adolescents, and adults. Lancet (London, England). (2017) 390(10113):2627–42. 10.1016/S0140-6736(17)32129-3 29029897 PMC5735219

[2] Obesity and overweight. Available at: https://www.who.int/news-room/fact-sheets/detail/obesity-and-overweight (cited 2022 Mar 24).

[3] Childhood Obesity Facts | Overweight & Obesity | CDC. Available at: https://www.cdc.gov/obesity/data/childhood.html (cited 2022 Mar 24).

[4] Vogel M Geserick M Gausche R Beger C Poulain T Meigen C Age- and weight group-specific weight gain patterns in children and adolescents during the 15 years before and during the COVID-19 pandemic. Int J Obes. (2021) 46(1):144–52. Available at: https://www.nature.com/articles/s41366-021-00968-2 (cited 2022 Mar 24) 10.1038/s41366-021-00968-2 PMC8458556 34556774

[5] Woolford SJ Sidell M Li X Else V Young DR Resnicow K Changes in body mass Index among children and adolescents during the COVID-19 pandemic. JAMA. (2021) 326(14):1434–6. 10.1001/jama.2021.15036 34448817 PMC8511973

[6] Campbell MK. Biological, environmental, and social influences on childhood obesity. Pediatr Res 2016 791. (2015) 79(1):205–11. 10.1038/pr.2015.208 26484623

[7] Sahoo K Sahoo B Choudhury AK Sofi NY Kumar R Bhadoria AS. Childhood obesity: causes and consequences. J Fam Med Prim Care. (2015) 4(2):187. Available at:/pmc/articles/PMC4408699/(cited 2022 Mar 24). 10.4103/2249-4863.154628 PMC4408699 25949965

[8] Reinehr T Hinney A de Sousa G Austrup F Hebebrand J Andler W. Definable somatic disorders in overweight children and adolescents. J Pediatr. (2007) 150(6):618–622.e5. Available at: https://pubmed.ncbi.nlm.nih.gov/17517246/(cited 2022 Mar 24) 10.1016/j.jpeds.2007.01.042 17517246

[9] Klöting N Blüher M. Adipocyte dysfunction, inflammation and metabolic syndrome. Rev Endocr Metab Disord. (2014) 15(4):277–87. Available at: https://pubmed.ncbi.nlm.nih.gov/25344447/(cited 2022 Mar 24) 10.1007/s11154-014-9301-0 25344447

[10] Scheja L Heeren J. The endocrine function of adipose tissues in health and cardiometabolic disease. Nat Rev Endocrinol. (2019) 15(9):507–24. 10.1038/s41574-019-0230-6 31296970

[11] Rutkowski JM Stern JH Scherer PE. The cell biology of fat expansion. J Cell Biol. (2015) 208(5):501–12. Available at: https://pubmed.ncbi.nlm.nih.gov/25733711/(cited 2022 Mar 24) 10.1083/jcb.201409063 25733711 PMC4347644

[12] Takemoto K Deckelbaum RJ Saito I Likitmaskul S Morandi A Pinelli L Adiponectin/resistin levels and insulin resistance in children: a four country comparison study. Int J Pediatr Endocrinol. (2015) 2015(1):1–12. 10.1186/1687-9856-2015-2 25904939 PMC4406215

[13] Must A Strauss RS. Risks and consequences of childhood and adolescent obesity. Int J Obes Relat Metab Disord. (1999) 23(Suppl 2):S2–11. Available at: https://pubmed.ncbi.nlm.nih.gov/10340798/(cited 2022 Mar 26) 10.1038/sj.ijo.0800852 10340798

[14] Vander Wal JS Mitchell ER. Psychological complications of pediatric obesity. Pediatr Clin North Am. (2011) 58(6):1393–401. 10.1016/j.pcl.2011.09.008 22093858

[15] Cheung N Wong TY. Obesity and eye diseases. Surv Ophthalmol. (2007) 52(2):180–95. Available at: https://pubmed.ncbi.nlm.nih.gov/17355856/(cited 2022 Feb 20) 10.1016/j.survophthal.2006.12.003 17355856 PMC2698026

[16] Hubbard LD Brothers RJ King WN Clegg LX Klein R Cooper LS Methods for evaluation of retinal microvascular abnormalities associated with hypertension/sclerosis in the atherosclerosis risk in communities study. Ophthalmology. (1999) 106(12):2269–80. Available at: https://pubmed.ncbi.nlm.nih.gov/10599656/(cited 2022 Apr 24) 10.1016/S0161-6420(99)90525-0 10599656

[17] Bhuiyan A Kawasaki R Lamoureux E Ramamohanarao K Wong TY. Retinal artery-vein caliber grading using color fundus imaging. Comput Methods Programs Biomed. (2013) 111(1):104–14. 10.1016/j.cmpb.2013.02.004 23535181

[18] Yau DPL Kim MM Tirsi DA Convit DA. Retinal vessel alterations and cerebral white matter microstructural damage in obese adolescents with metabolic syndrome. JAMA Pediatr. (2014) 168(12):e142815. Available at:/pmc/articles/PMC4420159/(cited 2021 Aug 24). 10.1001/jamapediatrics.2014.2815 25436854 PMC4420159

[19] Ho A Cheung CY Wong JS Zhang Y Tang FY Kam KW Independent and synergistic effects of high blood pressure and obesity on retinal vasculature in young children: the Hong Kong children eye study. J Am Heart Assoc. (2021) 10(3):1–11. 10.1161/JAHA.120.018485 PMC7955451 33496185

[20] Sousa-Sá E Zhang Z Pereira JR Wright IM Okely AD Santos R. Systematic review on retinal microvasculature, physical activity, sedentary behaviour and adiposity in children and adolescents. Acta Paediatr. (2020) 109(10):1956–73. Available at: https://pubmed.ncbi.nlm.nih.gov/31998981/(cited 2021 Aug 24) 10.1111/apa.15204 31998981

[21] Köchli S Endes K Infanger D Zahner L Hanssen H. Obesity, blood pressure, and retinal vessels: a meta-analysis. Pediatrics. (2018) 141(6):e20174090. 10.1542/peds.2017-4090 29743194

[22] Rijks J, Vreugdenhil A, Dorenbos E, Karnebeek K, Joris P, Berendschot T www.nature.com/scientificreports.

[23] Obradovic M Sudar-Milovanovic E Soskic S Essack M Arya S Stewart AJ Leptin and obesity: role and clinical implication. Front Endocrinol (Lausanne). (2021) 12:563. 10.3389/fendo.2021.585887 PMC8167040 34084149

[24] Siegrist M Hanssen H Neidig M Fuchs M Lechner F Stetten M Association of leptin and insulin with childhood obesity and retinal vessel diameters. Int J Obes. (2014) 38(9):1241–7. 10.1038/ijo.2013.226 24301134

[25] Van Aart CJC Michels N Sioen I De Decker A Nawrot TS De Henauw S. Associations of leptin, insulin and lipids with retinal microvasculature in children and adolescents. J Pediatr Endocrinol Metab. (2018) 31(2):143–50. 10.1515/jpem-2017-0374 29303782

[26] Hanssen H Siegrist M Neidig M Renner A Birzele P Siclovan A Retinal vessel diameter, obesity and metabolic risk factors in school children (JuvenTUM 3). Atherosclerosis. (2012) 221(1):242–8. 10.1016/j.atherosclerosis.2011.12.029 22244041

[27] Gishti O Jaddoe VWV Hofman A Wong TY Ikram MK Gaillard R. Body fat distribution, metabolic and inflammatory markers and retinal microvasculature in school-age children. The generation R study. Int J Obes. (2015) 39(10):1482–7. 10.1038/ijo.2015.99 26028060

[28] C-reactive protein, inflammation and coronary heart disease. Available at: https://www.infona.pl/resource/bwmeta1.element.elsevier-95d787dd-4ebe-39f1-88da-98d210102627 (cited 2022 Apr 24).

[29] Liccardo D Mosca A Petroni S Valente P Giordano U Mico’ AGA The association between retinal microvascular changes, metabolic risk factors, and liver histology in pediatric patients with non-alcoholic fatty liver disease (NAFLD). J Gastroenterol. (2015) 50(8):903–12. 10.1007/s00535-014-1024-1 25516385

[30] Bizzarri C Pedicelli S Romanzo A Bocchini S Bottaro G Cianfarani S The impact of IGF-I, puberty and obesity on early retinopathy in children: a cross-sectional study. Ital J Pediatr. (2019) 45(1):1–7. 10.1186/s13052-019-0650-x (cited 2021 Jul 4). 31029141 PMC6487055

[31] Cheung N Saw SM Islam FA Rogers SL Shankar A De Haseth K BMI And retinal vascular caliber in children. Obesity (Silver Spring). (2007) 15(1):209. Available at: https://pubmed.ncbi.nlm.nih.gov/17228049/(cited 2021 Aug 21) 10.1038/oby.2007.576 17228049

[32] Wang J Wang H Luo W Guo C Wang J Chen YE Leptin-induced endothelial dysfunction is mediated by sympathetic nervous system activity. J Am Hear Assoc Cardiovasc Cerebrovasc Dis. (2013) 2(5):e000299. 10.1161/JAHA.113.000299 PMC3835232 24042086

[33] Davis BM Crawley L Pahlitzsch M Javaid F Cordeiro MF. Glaucoma: the retina and beyond. Acta Neuropathol. (2016) 132(6):807–26. Available at: https://pubmed.ncbi.nlm.nih.gov/27544758/(cited 2022 Apr 24) 10.1007/s00401-016-1609-2 27544758 PMC5106492

[34] Jiang WJ Wu JF Hu YY Wu H Sun W Lu TL Intraocular pressure and associated factors in children: the shandong children eye study. Invest Ophthalmol Vis Sci. (2014) 55(7):4128–34. Available at: www.iovs.org (cited 2021 Aug 21) 10.1167/iovs.14-14244 24876285

[35] Pileggi C Papadopoli R De Sarro C Nobile CGA Pavia M. Obesity, blood pressure, and intraocular pressure: a cross-sectional study in Italian children. Obes Facts. (2021) 14(2):169–77. 10.1159/000514096 33794545 PMC8138192

[36] Aydemir GA Aydemir E Asik A Bolu S. Changes in ocular pulse amplitude and choroidal thickness in childhood obesity patients with and without insulin resistance. Eur J Ophthalmol. (2022) 32(4):2018–25. 10.1177/11206721211039337 34382437

[37] Baran R Baran S Toraman N Filiz S Demirbilek H. Evaluation of intraocular pressure and retinal nerve fiber layer, retinal ganglion cell, central macular thickness, and choroidal thickness using optical coherence tomography in obese children and healthy controls. Niger J Clin Pract. (2019) 22(4):539–45. 10.4103/njcp.njcp_471_18 30975960

[38] Akinci A Cetinkaya E Aycan Z Oner O. Relationship between intraocular pressure and obesity in children. J Glaucoma. (2007) 16(7):627–30. Available at: https://pubmed.ncbi.nlm.nih.gov/18091182/(cited 2021 Feb 22) 10.1097/IJG.0b013e318057528a 18091182

[39] Koçak N Arslan N Karti O Tokgoz Y Ozturk T Gunenç U Evaluation of the intraocular pressure in obese adolescents—minerva pediatrica. Minerva Medica—Journals. (2015) 67(5):413–8. PMID: . 26377780

[40] Bulus AD Can ME Baytaroglu A Can GD Cakmak HB Andiran N. Choroidal thickness in childhood obesity. Ophthalmic Surg Lasers Imaging Retin. (2017) 48(1):10–7. 10.3928/23258160-20161219-02 28060389

[41] Pekel E Altıncık SA Pekel G. Inner retinal thickness and optic disc measurements in obese children and adolescents. Arq Bras Oftalmol. (2020) 83(5):383–8. 10.5935/0004-2749.20200047 33084815 PMC12289277

[42] Pacheco-Cervera J Codoñer-Franch P Simó-Jordá R Pons-Vázquez S Galbis-Estrada C Pinazo-Durán MD. Reduced retinal nerve fibre layer thickness in children with severe obesity. Pediatr Obes. (2015) 10(6):448–53. 10.1111/ijpo.12005 25559237

[43] Hazar L Oyur G Yılmaz GC Vural E. Relationship of obesity and related disorders with ocular parameters in children and adolescent. Curr Eye Res. (2021) 46(9):1393–7. Available at: https://pubmed.ncbi.nlm.nih.gov/33586562/(cited 2022 Mar 18) 10.1080/02713683.2021.1884727 33586562

[44] Wong RL Zhao P Lai WW. Choroidal thickness in relation to hypercholesterolemia on enhanced depth imaging optical coherence tomography. Retina. (2013) 33(2):423–8. Available at: https://pubmed.ncbi.nlm.nih.gov/23089893/(cited 2021 Aug 30) 10.1097/IAE.0b013e3182753b5a 23089893

[45] Koca S Keskin M Saricaoglu MS Koca SB Duru N Şahin Hamurcu M Optic nerve parameters in obese children as measured by spectral domain optical coherence tomography. Semin Ophthalmol. (2017) 32(6):743–7. 10.1080/08820538.2016.1177095 27367416

[46] Karti O Nalbantoglu O Abali S Tunc S Ozkan B. The assessment of peripapillary retinal nerve fiber layer and macular ganglion cell layer changes in obese children: a cross-sectional study using optical coherence tomography. Int Ophthalmol. (2016) 37(4):1031–8. Available at: https://link.springer.com/article/10.1007/s10792-016-0371-8 (cited 2021 Aug 11) 10.1007/s10792-016-0371-8 27718081

[47] Demir S Özer S Alim S Güneş A Ortak H Yılmaz R. Retinal nerve fiber layer and ganglion cell-inner plexiform layer thickness in children with obesity. Int J Ophthalmol. (2016) 9(3):434–8. 10.18240/ijo.2016.03.19 27158616 PMC4844054

[48] Özen B Öztürk H Çatlı G Dündar B. An assessment of retinal nerve fiber layer thickness in non-diabetic obese children and adolescents. JCRPE J Clin Res Pediatr Endocrinol. (2018) 10(1):13–8. 10.4274/jcrpe.4810 28739552 PMC5838367

[49] Kurtul BE Cąkmak AI Elbeyli A Karaaslan A El Ç. Association of childhood obesity with retinal microvasculature and corneal endothelial cell morphology. J Pediatr Endocrinol Metab. (2020) 34(2):171–6. Available at: https://pubmed.ncbi.nlm.nih.gov/33544543/(cited 2022 Jan 30) 10.1515/jpem-2020-0483 33544543

[50] Nickla DL Wallman J. THE MULTIFUNCTIONAL CHOROID. Prog Retin Eye Res. (2010) 29(2):144. Available at:/pmc/articles/PMC2913695/(cited 2022 Apr 24). 10.1016/j.preteyeres.2009.12.002 20044062 PMC2913695

[51] Topcu-Yilmaz P Akyurek N Erdogan E. The effect of obesity and insulin resistance on macular choroidal thickness in a pediatric population as assessed by enhanced depth imaging optical coherence tomography. J Pediatr Endocrinol Metab. (2018) 31(8):855–60. 10.1515/jpem-2018-0079 29935116

[52] Erşan I Battal F Aylanç H Kara S Arikan S Tekin M Noninvasive assessment of the retina and the choroid using enhanced-depth imaging optical coherence tomography shows microvascular impairments in childhood obesity. J AAPOS. (2016) 20(1):58–62. 10.1016/j.jaapos.2015.10.006 26917074

[53] Kur J Newman EA Chan-Ling T. Cellular and physiological mechanisms underlying blood flow regulation in the retina choroid in health disease. Prog Retin Eye Res. (2012) 31(5):377. Available at:/pmc/articles/PMC3418965/(cited 2021 Aug 30). 10.1016/j.preteyeres.2012.04.004 22580107 PMC3418965

[54] Öztürk H Özen B Çatlı G Dündar BN. Macular variability in children and adolescents with metabolic syndrome: a cross-sectional study examining the associations with anthropometric measurements, metabolic parameters and inflammatory markers. JCRPE J Clin Res Pediatr Endocrinol. (2020) 12(1):63–70. 10.4274/jcrpe.galenos.2019.2019.0082 31434461 PMC7127882

[55] Teberik K Eski MT Doğan S Pehlivan M Kaya M. Ocular abnormalities in morbid obesity. Arq Bras Oftalmol. (2019) 82(1):6–11. 10.5935/0004-2749.20190007 30652762

[56] Agarwal A Saini A Mahajan S Agrawal R Cheung CY Rastogi A Effect of weight loss on the retinochoroidal structural alterations among patients with exogenous obesity. PLoS One. (2020) 15(7):e0235926. Available at:/pmc/articles/PMC7347179/(cited 2021 Aug 30) 10.1371/journal.pone.0235926 32645116 PMC7347179

[57] Celik G Gunay M Ozcabi B Gulturk U Kizilay O Vural A Evaluation of the impact of childhood obesity on retrobulbar hemodynamics and retinal microvasculature. European Journal of Ophthalmology. (2022) 32(6):3556–63. (cited 2022 Mar 18). 10.1177/11206721221086244 35243922

[58] Han S Leng Z Li X Yan W Shen S Liu L Retinochoroidal microvascular changes in newly developed obese children: an optical coherence tomography angiography study. BMC Ophthalmol. (2022) 22(1):1–6. Available at: https://bmcophthalmol.biomedcentral.com/articles/10.1186/s12886-022-02664-9 (cited 2022 Dec 18) 10.1186/s12886-021-02233-6 36384471 PMC9670671

[59] Dereli Can G Kara Ö Can ME. High body weight-related retinal vasculopathy in children with obesity. Eur J Ophthalmol. (2022) 32(2):1080–5. 10.1177/11206721211006570 33789499

